# Novel Therapeutic Options for Small Cell Lung Cancer

**DOI:** 10.1007/s11912-023-01465-7

**Published:** 2023-10-23

**Authors:** Stefania Canova, Benedetta Trevisan, Maria Ida Abbate, Francesca Colonese, Luca Sala, Alice Baggi, Sofia Paola Bianchi, Anna D’Agostino, Diego Luigi Cortinovis

**Affiliations:** 1 SC Medical Oncology, Fondazione IRCCS San Gerardo Dei Tintori, Monza, Italy; 2 Department of Medical-Surgical Specialties, University of Brescia, Radiological Sciences and Public Health, Brescia, Italy; 3 Radiation Oncology Department, Fondazione IRCCS San Gerardo Dei Tintori, Monza, Italy; 4 School of Medicine and Surgery, University of Milano Bicocca, Milan, Italy; 5 Medicine and Surgery Department, University of Milano Bicocca, Milan, Italy

**Keywords:** SCLC classification, Target therapy, Immunotherapy, PARP inhibitors

## Abstract

**Purpose of Review:**

The aim of this review is to focus on the recent advances in the molecular knowledge of small cell lung cancer (SCLC) and potential promising new treatment strategies, like targeting the DNA damage pathway, epigenetics, angiogenesis, and oncogenic drivers.

**Recent Findings:**

In the last few years, the addition of immunotherapy to chemotherapy has led to significant improvements in clinical outcomes in this complex neoplasia. Nevertheless, the prognosis remains dismal. Recently, numerous genomic alterations have been identified, and they may be useful to classify SCLC into different molecular subtypes (SCLC-A, SCLC-I, SCLC-Y, SCLC-P).

**Summary:**

SCLC accounts for 10-20% of all lung cancers, most patients have an extensive disease at the diagnosis, and it is characterized by poor prognosis. Despite the progresses in the knowledge of the disease, efficacious targeted treatments are still lacking. In the near future, the molecular characterisation of SCLC will be fundamental to find more effective treatment strategies.

## Introduction

Small cell lung cancer (SCLC) accounts for about 10–20% of all lung cancers and it is characterized by a high rate of proliferation, early metastases, and poor prognosis [1, 2]. As many as 98% of patients are smokers providing direct evidence that tobacco carcinogens are responsible for the initiation of SCLC [2, 3]. About 70% of patients have extensive disease (ED-) at diagnosis with a 2-year survival rate of approximately 2% [4]. For more than 30 years, chemotherapy (CT) with platinum and etoposide (PE) has been the standard front-line therapy [5, 6]. The introduction of immune checkpoint inhibitors (ICIs) set a new step in clinical practice for patients with ED-SCLC [7••].

As demonstrated by IMpower133 and CASPIAN, beyond others first-line trials conducted in asiatic population such as CAPSTONE-1 and ASTRUM-005, the addition of ICI to first-line platinum-based chemotherapy significantly improved overall survival (OS) with a reduction of 25 to 30% of the relative risk of death [7••, 8, 9••, 10]. Nevertheless, more research is needed to further improve this dismal benefit. Although SCLC is characterized by numerous genomic alterations, effective targeted therapies are still lacking. Indeed, over the past decade, the complete genomic profile of SCLC has highlighted a broad and complex genetic landscape of this tumour, including somatic mutations on transcription factors, receptor tyrosine kinases genes, and epigenetic changes in chromatin modifiers enzymes [2, 11–14]. However, all the trials to date have failed to demonstrate a survival benefit from using targeted agents. A deeper study of molecular aberrations could lead to the identification of new therapeutic targets [9••, 15].

## Toward a New Molecular Classification

Despite almost all cases of SCLC have total genomic loss of function of both TP53 and RB1, and are treated as a single disease, they are characterized by high heterogeneity [2]. Several authors have tried to classify SCLC into different subgroups based on molecular features.

Towards this end, Carney and colleagues identified in vitro two subtypes of SCLC, a classic (70%) and a variant subtype (30%). The classic phenotype cell lines express L-dopa decarboxylase, bombesin-like immunoreactivity, neuron-specific enolase, and the brain isozyme of creatine kinase. The variant subtype lack the expression of either L-dopa decarboxylase or bombesin-like immunoreactivity. The variant subtype is the most aggressive and is characterized by shorter doubling time and higher resistance to chemotherapy [16]. Furthermore, Poirier and colleagues identified two genes, achaete-scute homologue 1 (ASCL1) and neurogenic differentiation factor 1 (NEUROD1), both involved in neuroendocrine cells differentiation, but differentially expressed in SCLC. These genes are mutually exclusive in SCLC cell lines. ASCL1 has lower expression in variant SCLC compared to classic SCLC, and the authors proposed ASCL1 as the best candidate for differentiating SCLC subtypes [17]. ASCL1 and NEUROD1 regulate different genes, but ASCL1 is the leading cause of tumour formation. ASCL1 targets oncogenes such as MYCL1, SOX2, RET, and BCL2, suggesting it is required for SCLC survival. In addition, ASCL1 regulates NOTCH pathway genes, such as Delta-like ligand 3 protein (DLL3), which may represent a possible therapeutic target in SCLC. NEUROD1 targets the oncogene MYC, and cMYC protein expression is a predictive biomarker for multiple Aurora kinase inhibitors (AURKi). Therefore, ASCL1-positive and NEUROD1-positive SCLC cells have different origins and create two distinct subgroups of SCLC [18•].

Another gene identified in SCLC cells and potentially helpful to stratify SCLC into subgroups is the yes-associated protein 1 (YAP1). YAP1 is a key step in the tumour-suppressive Hippo pathway, which controls cell proliferation, apoptosis, and organ size. When Hippo signalling is active, YAP1 is inactivated and isolated in the cytoplasm for degradation. When Hippo signalling is inactive, YAP1 promotes pro-survival gene expression, proliferation, and tissue growth. RB1 is co-expressed with YAP1 in SCLC cell lines, thus it can be considered a surrogate of YAP1. Wild-type RB1 is present in about 25% of SCLC patients and is associated with decreased survival compared to patients with mutant RB1 [19].

POU class 2 homeobox 3 (POU2F3), also known as SKN-1a/OCT-11, is a transcription factor required for the generation of a chemosensory cell type of the gastrointestinal and respiratory tracts. POU2F3 is expressed in about 18% of SCLC and is mutually exclusive with ASCL1 and NEUROD1. Thus, POU2F3 identifies a unique subgroup of SCLC characterized by low expression of neuroendocrine markers. Interestingly, POU2F3-expressing cells are dependent on the tyrosine kinase receptor insulin-like growth factor 1 (IGF-1R) and therefore potentially sensitive to inhibitors of IGF-1R.

Based on these findings, Rudin et al. proposed a molecular classification of SCLC tumours. They identified 4 molecular subtypes called SCLC-A, SCLC-N, SCLC-Y and SCLC-P, according to the expression of transcription factors required for neuroendocrine (ASCL1 or NEUROD1) or non-neuroendocrine (YAP1 or POU2F3) differentiation. The last letters (in SCLC-?) indicate the transcription factor strongly associated with each subtype [20•].

Subsequent in vivo studies analysed the expression of ASCL1, NEUROD1, POU2F3 and YAP1 by immunohistochemistry (IHC). Baine’s research group observed that 69% of cases are ASCL1 dominant and 17% are NEUROD1 dominant. They confirmed that POU2F3 expression (7% of SCLC) is mutually exclusive of ASCL1 and NEUROD1. In addition, ASCL1/NEUROD1 double-negative tumours (14% SCLC) are a distinct subtype of SCLC characterized by low expression of neuroendocrine markers. Conversely, 7% of ASCL1/NEUROD1 double-negative cases have no identified transcription factors. Notably, YAP1 is absent or expressed at low levels in SCLC cells, compared with non-small cell lung cancer (NSCLC) cells where it is strongly expressed [21, 22••]. YAP1 protein expression seems also to correlate with the stage of disease, with highest expression in limited stage SCLC, an inflamed tumour microenvironment, and a better prognosis 23•]. Thus, it is not clear if YAP1-positive SCLC tumours represent a distinct subtype.

A similar study validated the molecular classification by IHC and showed intratumoral heterogeneity in SCLC tumours, which can contribute to chemo-resistance. In fact, Qu and colleagues observed that SCLC tumours can be positive for two (17.6%) or three (2.8%) subtype markers. Tumours that are predominantly positive for MYC are in either POU2F3 or YAP1 subgroups, while MYC negative tumours are mostly in ASCL1 and NEUROD1 subtypes. Moreover, tumour-associated CD8 + T cells are interconnected with molecular subtype; the non-ASCL1/NEUROD1 subtypes have significantly more CD8 + T cells than those of the ASCL1/NEUROD1 subtypes, thus they can benefit more from immunotherapy. Finally, Qu and colleagues also showed that a small percentage (6.3%) of SCLC tumours are negative for all four subtype markers [20•].

More recently, Gay and colleagues identified a SCLC subtype negative for all transcription factors, named SCLC-inflamed (SCLC-I). SCLC-I exhibits an inflamed phenotype that may make it more sensitive to immunotherapy. Indeed, it is characterized by high levels of immune checkpoints, MHC genes, and interferon-γ signalling pathway components and high levels of immune cell populations, such as T cells, natural killer cells, and macrophages [24].

**Fig. 1:**
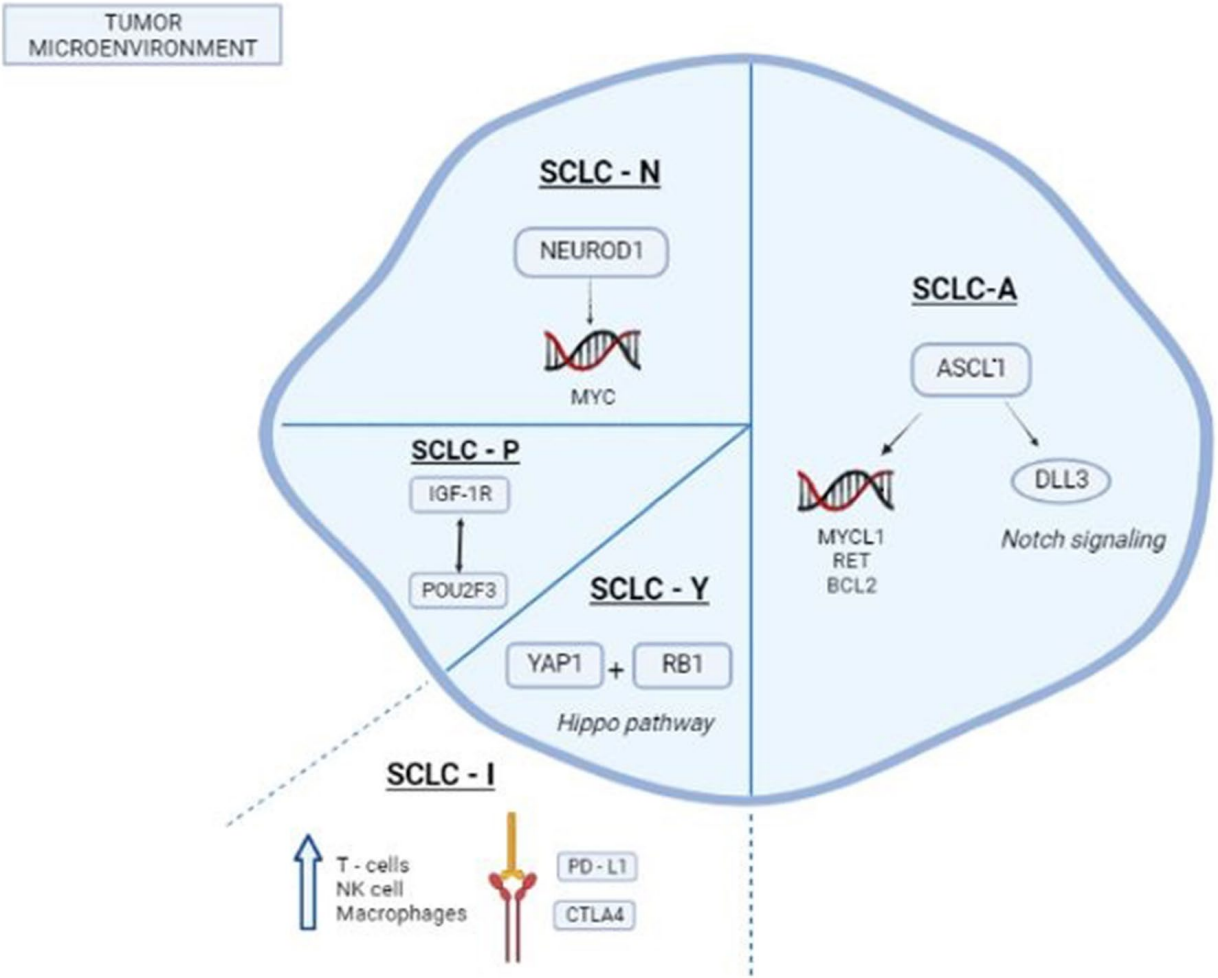
SCLC subtypes according to molecular features (SCLC-A, SCLC-N, SCLC-P, SCLC-Y) and Inflamed SCLC

Therefore, a molecular classification of SCLC represents the starting point to improve the current standard of care, overcome resistance to chemo-immunotherapy and to develop biomarker-guided therapies. The main characteristics of the molecular subtypes are reported in Fig. 1 and Table 1.

**Table 1:** SCLC molecular subtypes and main gene alterations

Molecular subtype	SCLC-A	SCLC-N	SCLC-Y	SCLC-P		SCLC-I
RB and TP53 loss of function						
Neuroendocrine differentiation		Non-neuroendocrine differentiation				
Gene alterations and Treatment targets	ASCL1	NEUROD1	YAP	POU2F3		No gene alterations
Upregulation						
MYCL1 SOX2 RET BCL2	MYC Arginine deprivation AURK A/B CHK1 LSD1 IMPDH	RB1 Arginine deprivation AURK A/B CHK1 IMPDH	IGF-1R Arginine deprivation AURK A/B CHK1 IMPDH		MHC INFY PATHWAY T cell-receptor genes	
Downregulation						
NOTCH signalling						
DLL3						

## Targeting the DNA Damage Repair Pathway

Interest in DNA damage and repair (DDR) has rapidly increased since it emerged that aggressive tumours, including SCLC, have a DDR pathway alteration.

Impairment in DDR pathway is a well-known predictive biomarker of platinum sensitivity. Furthermore, it is a predictive biomarker of ICIs benefit due to its positive correlation with tumour mutation burden (TMB).

There are five major DNA damage repair pathways. The base excision repair (BER) mechanism repairs single strand breaks. The homologous recombination repair (HRR) and the non-homologous end joining (NHEJ) fix double-strand breaks. The mismatch repair (MMR) mechanism repairs replication errors. Finally, the nucleotide excision repair (NER) repairs platinum and UV radiation damage [25, 26].

## PARP Inhibitors

Poly (ADP-ribose) polymerase (PARP) inhibitors target a family of proteins called PARP involved in several processes including DNA repair (using BER, HRR and NHEJ) and apoptosis. PARP1 and PARP2 are key proteins that are activated when DNA damage occurs, which they detect and then send signals to other proteins to repair it.

There are tumours in which one of the DNA repair pathways, HRR, is no longer functional due to mutations in one of its main components, such as BRCA1 or BRCA2 proteins. Therefore, the DNA damage, originating from the genetic instability of the tumour, can neither be repaired either by PARP- 1 nor inhibited by the drug, or by the alternative pathway of homologous recombination, whose functionality is intrinsically compromised in these types of tumours. The simultaneous mutation (or inhibition) of a pair of genes or biochemical pathway, unlike the mutation (or inhibition) of only one of these, causes cell death and is called synthetic lethality.

SCLC is sensitive to DNA damage, and PARP1 is highly expressed in SCLC. Combination strategies with PARP inhibitors (PARPis) have been studied previously in second line, and then in first line, with only modest results.

Following an initial phase I/II study that established the recommended phase II study dose of olaparib in combination with temozolomide (TMZ), a phase II study was conducted to evaluate clinical activity in relapsed SCLC. The combination demonstrated a 41.7% overall response rate (ORR), median progression-free survival (PFS) of 4.2 months and median overall survival (OS) of 8.5 months [27].

Likewise, a phase II study evaluated the combination of veliparib and TMZ versus placebo and TMZ (1:1) in recurrent SCLC. It showed an increased ORR (39% v 14%; p = 0.016), but neither an increase in PFS (*p* = 0.19) nor in median OS (8.2 months versus 7.0 months; *p* = 0.50). Moreover, grade 3/4 thrombocytopenia and neutropenia were more common in the combination arm. Notably, a statistically and clinically significant improvement of PFS (5.7 v 3.6 months; *P* = 0.009) and OS (12.2 v 7.5 months; *P* = 0.014) was observed in patients with SLFN11-positive tumours treated with veliparib plus TMZ [28].

Based on these results, PARPis were tested in earlier lines.

Olaparib was evaluated as a maintenance monotherapy in patients with SCLC in partial or complete response following first-line treatment or chemo-radiation therapy. Patients were randomized 2:2:1: olaparib 300 mg twice a day (BD), olaparib 200 mg three times a day (TDS), placebo BD, or placebo TDS. There was no significant difference in either PFS or OS between olaparib and placebo [29].

First-line veliparib was tested in combination with cisplatin plus etoposide chemotherapy in patients with ED-SCLC. Patients were randomized 1:1: chemotherapy plus veliparib versus chemotherapy plus placebo. The experimental combination showed a significant benefit in PFS (median PFS 6.1 vs 5.5 months, observed stratified PFS HR 0.63, one-sided *P* = 0.01), but neither in OS nor ORR. No predictive biomarkers were identified [30].

Conversely, in another study SLFN11 was identified as a potential predictive biomarker of benefit from PARPis in patients with untreated ED-SCLC. In this phase 2 study, randomization was 1:1:1: veliparib plus chemotherapy with carboplatin plus etoposide (CE) followed by veliparib maintenance, veliparib plus CE followed by placebo or placebo plus CE followed by placebo (control arm). The first arm combination improved PFS compared with the control arm (HR 0.67; 80% confidence interval (CI). 0.50–0.88; *p* = 0.059), although the difference was not clinically significant (median PFS 5.8 versus 5.6 months), with a trend in SLFN11 positive patients (HR, 0.6; 80% CI: 0.36–0.97). Moreover, there was no significant benefit in OS [31].

Another PARPi, niraparib, was tested as a maintenance strategy in Asian patients with ED-SCLC with partial or complete response to first line chemotherapy in a randomized, double-blind, phase 3 study. Likewise, niraparib showed a statistically significant improvement in PFS (HR 0.66; 95% CI: 0.46–0.95; *p* = 0.0242), even though clinically modest (median PFS 1.54 versus 1.36 months) [32].

Therefore, despite the fact that the DDR pathway represents a potential therapeutic target, DDR alterations seem not to correlate with platinum chemotherapy outcomes in SCLC, in contrast to ovarian or breast cancers.

Another area of research is the use of PARPi to overcome ICI resistance. Preclinical studies demonstrated that inhibition of DDR proteins such PARP enables the anti-tumour immune response of PD-L1 inhibition through T cell-mediated effects. These pre-clinical studies provided a rationale for combining PARP inhibitors with immunotherapies in SCLC [33, 34]. A phase 2 study of durvalumab plus olaparib in patients with relapsed SCLC did not meet its primary endpoint of PFS, but it did demonstrate the importance of appropriate patient selection on the basis of tumours and tumor microenviroment (TME) biological characteristics [35]. Several trials studying combination of PARP inhibitor plus ICIs are ongoing, for both pre-treated ED-SCLC (phase 2 NCT04701307, phase 1/2 NCT04728230), and for the maintenance/consolidation therapy following first-line treatment (phase 2 NCT04782089, phase 2 NCT04334941, phase 1b/2 NCT03830918).

### Lurbinectedin

Lurbinectedin is a drug that prevents oncogenic transcription activity in cancer cells. It inhibits RNA formation by preventing the binding of transcription factors to their promoters, thus promoting DNA double-strand breaks and inducing cell death [36, 37]. Moreover, lurbinectedin reduces inflammation of the tumour microenvironment and decreases transcription within tumour-associated macrophages. This induces tumour cell death, reduces angiogenesis, and enhances anti-tumour immunity [38]. Lurbinectedin showed increased beneficial activity in tumours with defects in DNA mismatch repair, including SCLC.

Initially, a phase II single-arm basket trial evaluated patients with ED SCLC pre-treated with only one line of treatment. Patients were treated with lurbinectedin 3.2 mg/m^2^ every 3 weeks until disease progression or unacceptable toxicity. An ORR of 35.2%, median PFS of 3.5 months and OS of 9.3 months were reported [39]. Based on these results, lurbinectedin has been granted by FDA as orphan drug status for the treatment of patients that progress after first-line platinum-based chemotherapy.

Moreover, combination strategies were tested to improve efficacy. A phase I study investigated the association of lurbinectedin 2.0 mg/m^2^ and doxorubicin 40 mg/m^2^. The combination achieved an ORR of 91.7% in second-line patients with sensitive disease (platinum-free interval ≥ 90 days) and an ORR of 33.3% with resistant disease (platinum-free interval < 90 days). In third-line setting patients achieved an ORR of 20% in all subgroups [40].

Following these encouraging results, the Phase III study ATLANTIS tested the same combination compared to topotecan or CAV (cyclophosphamide, doxorubicin, and vincristine) in over 600 patients progressing to first-line platinum-containing chemotherapy. The combination did not improve OS compared to the control arm. Furthermore, an OS difference was not observed in either patients with or without central nervous system involvement. Despite this, the combination showed a better toxicity profile compared to the control arm [41].

### Targeting Angiogenesis

The angiogenesis process is crucial for tumorigenesis and remains active during cancer growth. The vascular endothelial growth factor (VEGF) pathway is over-active in a lot of tumours, including SCLC [42]. It has been demonstrated that the hypoxia-inducible factor 1a (HIF-1a) can promote SCLC growth and angiogenesis and, therefore, be another potential therapeutic target [43].

Bevacizumab is a humanised monoclonal antibody targeting VEGF, approved for several malignancies, such as colon-rectal, kidney, and ovarian cancer [44]. Bevacizumab is the most studied antiangiogenic therapy in SCLC, with inconsistent results.

In metastatic SCLC a number of studies have been conducted, mainly phase II studies.

Regarding relapsed pre-treated-patients, bevacizumab was evaluated in combination with chemotherapy (topotecan or paclitaxel) in two phase II studies. Results showed good tolerability, but few efficacy advantages compared to historical controls [45, 46].

Recently, a phase I study evaluating lurbinectedin plus paclitaxel with or without bevacizumab in advanced solid tumours, including SCLC, showed no major interactions between the drugs [47].

Most of the trials evaluated bevacizumab in combination with chemotherapy in a first line setting. Two phase II studies focused on the combination of a platinum agent plus irinotecan and bevacizumab, which showed similar results. In fact, median PFS was 7 months (95% CI, 6.4–8.4 months) with cisplatin, irinotecan and bevacizumab, and median time to progression was 9.13 months (95% CI, 7.36–9.46 months) with carboplatin, irinotecan and bevacizumab. Likewise, median OS was 11.6 months (95% CI, 10.5–15.1 months) and 12.1 months (95% CI, 9.6–13.5 months), respectively [48, 49]. Bevacizumab was also evaluated in the first line maintenance setting in the phase II studies showing an improvement in PFS and OS compared to historical controls for those patients who received cisplatin and etoposide without bevacizumab maintenance [50]. The SALUTE trial was the first placebo-controlled, double-blind, randomized phase II trial that assessed bevacizumab in this therapy setting. It showed a small PFS improvement (5.5 months in the Bevacizumab arm versus 4.4 months in the placebo arm, HR 0.53) without an OS advantage [51].

More recently, two phase III trials have been conducted to evaluate the efficacy of adding bevacizumab to chemotherapy as first-line treatment. They confirmed a favourable toxicity profile of the combination, although without either a clinically significant PFS or an OS improvement [52, 53].

Regarding limited-disease SCLC, only one phase II study investigating the activity of bevacizumab as maintenance therapy after chemo-radiotherapy has been conducted, and reported a median OS of 15 months, ORR and 2-year PFS of 80% and 54% respectively [54]. However, safety represents a concern for the high incidence of tracheoesophageal fistulae.

Pazopanib is an orally administered tyrosine kinase inhibitor (TKI) targeting vascular endothelial growth factor receptor (VEGFR), platelet-derived growth factor receptors (PDGFR), and c-kit. It has been studied in SCLC as maintenance therapy following platinum and etoposide chemotherapy, in the study KCSG-LU12-07, which showed a statistically significant PFS improvement (3.7 months in pazopanib group versus 1.8 months in the placebo group, *p* < 0.0001). However, pazopanib did not show a good toxicity profile with 51.2% interruption rate due to adverse events [55]. Pazopanib has also been evaluated in second line of therapy by the Hellenic Oncology Research Group. In this phase II study, patients with platinum-sensitive and platinum-resistant SCLC were enrolled and received pazopanib. Albeit small, the study showed promising activity in patients with platinum-sensitive disease, with a DCR of 48.3% [56].

Sunitinib is an oral TKI that binds VEGFR, PDGFR, Flt-3, and Kit [44]. As with pazopanib, sunitinib has been studied as maintenance therapy following CT in SCLC in two trials. In a phase II, non-randomized trial, it was evaluated following CT with irinotecan and carboplatin, and showed a 1-year OS of 54% with rare adverse events [57]. In the CALGB 30504 a randomized, placebo-controlled, phase II trial, patients were randomized to receive sunitinib or a placebo after etoposide plus platinum-based chemotherapy. Sunitinib was found to be safe and effective. Indeed, median PFS was 2.1 months for placebo and 3.7 months for sunitinib arm (*p* = 0.02) [58].

Nintedanib is a potent TKI that links to VEGFR1-3, FGFR 1–3, and PDGFR α and β [59]. A phase 2 study was conducted to evaluate its efficacy and safety in relapsed/platinum-refractory SCLC. Nintedanib had a manageable toxicity profile but very poor efficacy in these patients’ setting. Indeed, ORR was 5%, with 1 month of PFS [59].

Anlotinib is an orally administered TKI directed to VEGFR, fibroblast growth factor receptor (FGFR), PDGFR, and c-kit [44]. Several studies have been developed in China, where anlotinib is approved for patients with pre-treated SCLC to evaluate the safety and the efficacy of this drug [44]. It has been studied alone as a third or fourth line of treatment [60], and in combination with standard CT as a first line of therapy [61, 62], showing good tolerability and promising clinical efficacy.

Another drug studied mainly in China is apatinib, a VEGF2 inhibitor. Likewise, some studies evaluating apatinib as maintenance or in second and further line therapies have been developed. As with anlotinib, apatinib showed a safe toxicity profile and interesting clinical activity [63–66].

## Targeting Proteins and Oncogenic Drivers

Oncogenic drivers are genes with acquired mutations that are causally linked to cancer initiation and progression [67]. In NSCLC, a number of oncogenic driver mutations have been determined, enabling a molecular targeted treatment approach. Conversely, in SCLC the main driver mutations are loss of function of suppressor genes such as RB1 (60–90%) and TP53 (75–90%) [11, 68]. RB and TP53 mutations lose function and as a result are un-targetable. Nevertheless, a few activating mutations have been identified in SCLC, introducing the initial concept of “oncogene-addiction” in this aggressive tumour.

### DLL-3

DLL-3 (delta-like protein 3) is a cell surface protein that inhibits the tumor suppressor gene NOTCH-1 and consequently upregulates the expression of Achaete-scute homolog 1 (ASH-1), a transcription factor driving SCLC oncogenesis [69]. DLL-3 is highly expressed in SCLC and other high-grade neuroendocrine tumours but not in normal lung tissue, suggesting that this protein may play an important role in neuroendocrine tumorigenesis and can be a vector for delivering cytotoxic agents to DLL3-positive cells [70–72]. Rovalpituzumab tesirine (Rova-T) is a first-in-class, antibody–drug conjugate (ADC) composed of an IgG1 monoclonal antibody that targets DLL3 linked to a toxic DNA agent, pyrrolobenzodiazepine (PBD), and a protease-cleavable linker [73]. Phase I and phase I–II studies have been conducted to assess safety and pharmacokinetics [74–76]. These seemingly promising results led to the development of phase II trials, like the Trinity Study, in which DLL-3 high SCLC demonstrated a 14.3% ORR with a median OS of 5.7 months [77]. These disappointing results have since been confirmed by a few Phase III trials. The THAOE study, a randomized trial, compared Rova-T with topotecan in second-line therapy in DLL3-high metastatic SCLC, but did not show any benefits in terms of OS [78]. Likewise, the MERU trial, a phase III study evaluating Rova-T in maintenance therapy after first-line platinum chemotherapy did not meet the primary endpoint. Thus, the lack of survival benefit led to an early discontinuation of the study. Moreover, Rova-T was associated with higher, unique, and unacceptably adverse events (AEs) like pleural and pericardial effusions, peripheral oedema and photosensitivity [79]. Therefore, the development of Rova-T has been discontinued. Nevertheless, new generation anti-DLL-3 drugs are currently under investigation. AMG 757 (tarlatamab) is a first-in-class bispecific T cell engager that binds DLL-3 and CD3 domains of the T cell receptor, leading to T cell-mediated tumour lysis. In vitro, an interesting killing activity has been shown, which led to the development of Phase I trials, currently ongoing [80–82]. Moreover, a phase II trial (NCT05060016) is evaluating safety and efficacy in the pre-treated SCLC population. Another drug, AMG 119, a chimeric antigen receptor T cell (CAR-T), showed promising results in vitro and in vivo, but the Phase I trial has been suspended for the time being [80].

### CD56

CD56, also known as NCAM1, is an adhesion molecule involved in nervous system differentiation and immune surveillance. CD56 aberrant expression is evident in many solid tumours with neuroendocrine origin, like SCLC. For this reason, it could be an appropriate molecular target [83]. Lorvotuzumab mertansine (LM, IMGN901) is an ADC with an anti-CD56 antibody linked to a microtubule inhibitor, DM1 [69]. A phase I–II study evaluated LM in association with carboplatin/etoposide regimen in ED-SCLC, but this showed no evidence of efficacy improvement in the combination regimen as well as resulting in an increased number of AEs. In fact, 21 patients (63.6%) had a treatment-related adverse event (TRAEs) resulting in death [84]. Another Phase I study showed 96.9% of TRAEs, mostly grade 1 or 2 [83]. Therefore, further investigations are needed to better understand safety and efficacy of LM.

### TROP 2

TROP2 (trophoblast cell surface antigen-2) is a transmembrane glycoprotein member of the EpCAM family, overexpressed in many tumours (e.g., breast cancer and NSCLC), acting either like an oncogene driver or an onco-suppressor. Non-tumour-tissues rarely express TROP2, so it can be a reliable therapeutic target [69]. Sacituzumab govitecan (SG) is a Trop2-directed antibody linked to SN-38, an active metabolite of irinotecan [85, 86]. The IMMU-132–01 phase I–II basket trial evaluated the efficacy and safety of SG in several tumours, including sixty-two SCLC patients. In this population, an ORR of 17.7% was shown, with a median OS of 7.1 months (95% CI, 5.6–8.1 months) and a median PFS of 5.5 months (95% CI, 3.6–7.6 months) [86]. A phase I–II clinical trial in previously treated ED-SCLC patients showed an ORR of 14%, a median OS of 7.5 months (95% CI, 6.2–8.8) and a median PFS of 3.7 months (95% CI, 2.1–4.3) [87]. These results are encouraging but need to be confirmed in larger prospective studies. Trials evaluating SG in combination with PARP inhibitors are currently ongoing [69].

### SOX2

SOX2 is a pluripotency factor and a key regulator of neuroendocrine cells. A SOX2 amplification, regulated by the Hedgehog cascade, is associated with SCLC growth [11]. A phase I trial of sonidegib (Hedgehog inhibitor) in combination with cisplatin and etoposide for ED-SLCLC treatment showed an ORR of 79% (95% CI, 49–95%) and 6/15 TRAEs [88]. Another study evaluating vismodegib, another Hedgehog inhibitor, in combination with cisplatin and etoposide did not show any benefit in terms of either OS or PFS [89].

### Bcl-2

Bcl-2 is an anti-apoptotic protein frequently overexpressed in SCLC [90]. A phase I and a phase II study with navitoclax, a selective inhibitor of Bcl-2 and Bcl-x, showed limited activity in advanced and recurrent SCLC with a high rate of serious thrombocytopenia [91, 92]. To mitigate this relevant side effect navitoclax was re-engineered as venetoclax. Venetoclax selectively binds Bcl-2 without causing thrombocytopenia, and showed promising preclinical results [90, 93]. A phase II study with another Bcl-1 inhibitor, obatoclax, in addition to carboplatin and etoposide demonstrated a good safety profile, but no clinical benefit in terms of either ORR, OS or PFS [94]. Similarly, AT-101 is an oral, pan-Bcl2 inhibitor, evaluated in a phase I-II trial in combination with topotecan. The combination is safe, but no clinical benefit was achieved in the early phases, so the enrolment closed [95].

### AURK

AURKs (aurora kinases) are a family of kinases that play a key role in the cell cycle, in particular in the cell duplication. Aurora kinase A (AURKA) promotes mitosis through activation of checkpoint kinase 1 (CHK1), and is highly expressed in SCLC, thus representing a potential therapeutic target. An AURK hyperexpression is a pro-tumorigenic pathway in many cancer types, including SCLC. For this reason, a number of AURK inhibitors (AURK-is) have been developed in the last few years [96]. AURK-is action mechanism has been tested in mice [97], and this led to Phase I and Phase II clinical trials in pre-treated patients. Alisertib is an oral AURKA inhibitor that, as a single agent, showed beneficial activity in a phase II trial in terms of ORR (21%, 10/48 pts) in relapsed or refractory SCLC [98]. Following these encouraging findings, alisertib was studied in combination with weekly paclitaxel compared to placebo plus weekly paclitaxel in relapsed or refractory SCLC. The combination proved a PFS advantage (HR 0.77, 95% CI: 0.557–1.067, *p* 0.113), while no benefit in OS was observed. Interestingly, as previously reported in another study [99], Myc was revealed to be a potential predictive biomarker. In fact, although the number of patients is small (33), a greater advantage for PFS was observed in patients with c-Myc expression (4.64 vs 2.27 months, HR 0.29, 95% CI: 0.12–0.72) compared to c-Myc-negative patients [100].

Danusertib, a multi AURK-i, was tested in 24-h infusion in solid tumours in a Phase I study. It included 2 SCLC patients of which one had an ORR of 23 weeks [101]. A Phase II trial with danusertib showed a median PFS of 8.1 weeks (95% CI, 7.1–8.9) and a median OS of 11.4 months (95% CI, 4.5–n.r.) in the SCLC cohort [102].

Finally, prexasertinib, a selective CHK1 inhibitor, was investigated as a single-agent in a phase II trial in patients with pre-treated ED-SCLC, but it failed to demonstrate beneficial activity [103].

### MYC

MYC gene amplifications have been identified in 6–25% of SCLC, especially in the SCLC-N subtype, and are associated with poorer outcomes and treatment resistance [97]. Indeed, data suggests that MYC promotes a subset of more aggressive SCLC subtype with lower expression of neuroendocrine markers [96]. A preclinical trial showed that arginine depletion with pegylated arginine deiminase (ADI-PEG 20) suppresses tumour growth in mice that had MYC-driven tumours [99]. A Clinical trial to evaluate ADI-Peg 20 is ongoing (NCT03449901). A few cases have been described in which MYC amplification can make cells sensitive to AURK-is [104], and this is supported by preclinical data [97, 105].

## Targeting Epigenetics

Epigenetic refers to modulation of gene expression profiling without alteration of the DNA sequence [106]. Several studies demonstrated a critical role of epigenetic alterations during SCLC development and progression [107–111].

DNA methylation is one of the epigenetic processes, and it is involved in key SCLC genes’ regulation [112–114].

DNA methyltransferases (DNMTs) are the effectors of this process, and DNMTs inhibitors are small molecules that when used in low doses, can induce the expression of silenced genes, while when used in high doses can directly kill cancer cells [115, 116].

Another important epigenetic mechanism consists of histone modifications, in particular methylation and acetylation. Histone methyltransferases (HTMs) and histone demethylases (HDMs) are the effectors of histone methylation, while acetylation is regulated by histone acetyltransferases (HATs) and histone deacetylases (HDACs) [117]. A few studies showed that these mechanisms are a cause of SCLC pathogenesis and development and of chemotherapy sensitivity/resistance [118–121].

Several inhibitors of histone modifications have been tested for SCLC treatment. When considering HDAC inhibitors, preclinical studies demonstrated that different molecules suppress SCLC cell proliferation and promote chemotherapy anti-tumour effects in SCLC cell lines [122–126]. Many phase I/II trials are currently ongoing. Furthermore, little, and inconsistent data is available. One single-centre phase I trial showed that the combination of the HDAC inhibitor belinostat with cisplatin plus etoposide is safe and active in SCLC and other neuroendocrine cancers [127]. Nevertheless, a phase II trial evaluating the HDAC inhibitor panobinostat and a phase II trial investigating the HDAC inhibitor romidepsin showed a lack of activity of these two compounds [128, 129].

Also, HMTs and HDMs inhibitors showed activity against SCLC lines in preclinical studies. For example, the HMT enhancer of zeste homolog 2 (EZH2) is highly expressed in SCLC, and preclinical evidence showed that its inhibition can overcome SCLC chemoresistance [130]. At present no data derived from clinical trials is available for EZH2 inhibitors.

Lysine specific demethylase-1 (LSD1) is a highly expressed HDM in various haematological and solid tumours, including SCLC [131]. Its inhibitors, ORY-1001 (Iadademstat) and GSK2879552, repressed SCLC tumorigenesis and growth in preclinical studies [132, 133]. At present the findings from clinical studies are discouraging and inconsistent with pre-clinical analysis. A phase I multicentre, open-label study that investigated the safety, pharmacokinetics, pharmacodynamics, and clinical activity of the LSD1 inhibitor GSK2879552 in patients with relapsed or refractory SCLC was terminated due to a high incidence of adverse events and poor disease control [134]. Nevertheless, there is a lot of interest in epigenetic drugs, in particular LSD-1 inhibitors, and many phase I or II clinical trials are ongoing (e.g., NCT05420636, NCT03850067, NCT05268666, NCT03460977).

Finally, multitarget epigenetic molecules are also currently under investigation. Very recently, JBI-802 has been identified as a dual LSD1/HDAC6/8 inhibitor, which displayed huge antiproliferative effects in SCLC and others haematological and solid tumours [135]. Moreover, combinations with DNMT and HDAC inhibitors are being researched only in preclinical settings to date [136].

Therefore, all this data underlines the crucial role of epigenetics in carcinogenesis and proliferation of tumor cells.

Furthermore, recent studies have demonstrated the crucial role of epigenetic machinery in modulating immune cell functions and antitumor immune response, modifying tumour immunogenicity as well as affecting immune cells [137].

Epigenetic modifications regulate the antigen processing and presentation, the maturation and differentiation of dendritic cells, the activation, trafficking and infiltration of T cells, and the development of Treg [137, 138]. Moreover, it is demonstrated that the expression of PD-1 and PD-L1 in SCLC is partly related to an upregulation of DNA methyltransferase 1 (DNMT1) [139].

Considering in particular the antigen presentation phase, preclinical data showed that SCLC has a low intrinsic expression of MHC class I and II molecules [140, 141], and one of the underlying mechanisms behind this consists of epigenetic modifications, as demonstrated by the restoration of MHC class I expression as well as the T cell-mediated killing of tumour cells due to the pharmacological inhibition of EZH2 in SCLC cell lines [142].

Conversely, considering the lack of function of T cells in the TME, recent studies found that DNMT inhibitors and histone modifications inhibitors can reverse tumour immune evasion. This is in addition to being able to modulate T cell exhaustion state towards effector and memory T cell phenotypes in mouse models of NSCLC, ovarian cancer and melanoma cell lines, thus sensitizing to anti-CTLA4 and anti-PD1 therapy [143–145]. In this context, the LSD1 HDM plays a critical role due to its ability to suppress endogenous double stranded RNA (dsRNA) levels and interferon (IFN) responses in tumour cells, as demonstrated by a preclinical study that showed that LSD1 inhibition in tumour cells causes intracellular dsRNA stress and resultant IFN activation and anti-tumour T cell immunity promotion. Moreover, the authors demonstrated that LSD1 depletion converts tumours resistant to PD-1 blockade to cells responsive to ICIs [146]. Based on these findings, clinical trials on LSD1 inhibitors in combination with ICIs are ongoing for various solid tumours, including SCLC. A phase I/II trial is evaluating bomedemstat in combination with maintenance atezolizumab for ED-SCLC in first-line following induction chemo-immunotherapy (NCT05191797). A phase IImulti-cohort study is ongoing to assess safety and efficacy of CC-90011, an oral LSD1 inhibitor, in combination with nivolumab in pre-treated SCLC patients (NCT04350463).

Epigenetic mechanisms also have a role in the evasion of innate immunity. In particular, it has been demonstrated that SCLC aggressiveness and metastasis is partially related to a low expression of NK-activating ligands (NKG2DL) due to an epigenetic silencing with consequent loss of NK cell recognition. Restoring NKG2DL using HDAC inhibitors in preclinical models suppressed tumour growth and metastasis by inducing infiltration and activation of NK and T cells [147]. Research on the potential role of these epigenetic drugs to overcome ICI resistance is at a very early stage, but expectations are high for the future.

## Overcoming the ICI Resistance

After more than 20 years without innovation in SCLC management, ICIs targeting PD-1/PD-L1 axis have changed treatment algorithm for this disease [7••, 9••]. Moreover, there is a compelling rationale for ICI efficacy based on the high immunogenicity of SCLC related to a high genomic instability and consequent high tumour mutational burden (TMB) [148]. Nevertheless, ICI efficacy remains modest, and there is a significant focus on overcoming the ICI resistance in SCLC in first-line and relapsed settings.

The main strategy to increase ICI therapeutic activity consists of combinations of different immunotherapy approaches, which co-target immune molecules highly expressed in SCLC. Indeed, recent studies on SCLC biology showed a low expression of PD-L1 and tumour-infiltrating lymphocytes (TILs) in SCLC samples in contrast to a higher expression of other immune inhibitory proteins, such as B7-H3, the T cell immunoreceptor with immunoglobulin and ITIM domain (TIGIT), the T cell immunoglobulin mucin receptor 3 (TIM3), the lymphocyte activation gene 3 (LAG3), and others [149, 150].

### TIGIT

TIGIT is transmembrane protein expressed by a variety of immune cells that, when activated, induces a tolerogenic microenvironment. In particular, it competes with CD226 (a transmembrane protein that enhances lymphocytes cytotoxicity mechanisms) on activated T cells for binding to CD155, CD112, and CD113 ligands, and it also acts on Natural Killer cells. Moreover, in various cancers, the TIGIT expression profile correlates with the expression of other immune inhibitors receptors, including LAG3, CTLA4, and PD-16–7. Anti-TIGIT monoclonal antibodies are tested in several ongoing trials, which have enrolled patients affected by various solid tumors. Considering limited-stage SCLC, a phase II-3-arm study (NCT04952597) is examining the combination of the anti-TIGIT monoclonal antibody ociperlimab (BGB-A1217) plus tislelizumab (anti-PD1) concurrent with chemoradiotherapy (CRT). Another phase II study (NCT04308785) is evaluating atezolizumab with or without the anti-TIGIT tiragolumab as consolidation therapy following CRT.

Turning to untreated ED-SCLC, the phase III SKYSCRAPER-02 (NCT04256421) is ongoing: 490 patients have been enrolled and randomized to receive standard care (atezolizumab plus carboplatin and etoposide) either with or without tiragolumab. Unfortunately, at the interim analysis at a median follow up of 14.3 months this study failed to meet its co-primary endpoints of PFS improvement (median PFS was 5.4 months (95% CI 4.7–5.5) with tiragolumab vs 5.6 months without this agent). Nevertheless, the study will continue as planned until final OS analysis [151, 152].

### LAG-3

LAG-3 is an inhibitory immunoreceptor expressed on immune cells including activated T, T-regulatory, NK. and plasmacytoid dendritic cells. Its best-characterized ligand is the major histocompatibility complex class II (MHC-II), but there are other ligands, such as fibrinogen-like protein 1 (FGL-1). Sustained T cell activation in a chronic inflammatory environment, such as TME, increases LAG-3 co-expression with co-inhibitory receptors including PD-1 and, when activated, LAG-3 contributes to T cell suppression and subsequent immune dysfunction [153]. Several LAG-3–targeting molecules are currently in early stages of clinical development with early results suggesting a modest benefit when used as single agents, but dual LAG-3/PD-1 blockade has a significant role in reducing tumour growth by increasing the proportion of effector T cells in the tumour [154, 155].

To date, patients with SCLC have been recruited only in basket trials such as a phase 1/2 study evaluating LAG525 (anti-LAG3) in combination with spartalizumab (anti-PD-1). This SCLC cohort met the criteria for expansion on the basis of the clinical benefit rate [156]. The ongoing phase 1 trial is evaluating tebotelimab, which is a monoclonal antibody engineered to bind PD-1 and LAG-3 concomitantly or independently (NCT03219268).

### TIM-3

TIM-3 is an inhibitory receptor with a crucial role in both innate and adaptive immune responses, and it is often co-expressed with PD-1 [156, 157]. TIM-3 also has a role in resisting PD-1 blockade, and preclinical studies demonstrated that dual TIM-3 and PD-1 blockade is more effective than targeting either pathway alone [158, 159]. Further, Anti-TIM3 drugs, like the anti-LAG3 drugs mentioned above, are at a very early stage of development. At the moment, the available data for SCLC patients derives from the small numbers of a phase 1 basket trial [160], so it will still take a long time to understand the real potential of these treatments.

Other strategies to overcome ICI resistance include combinations of ICIs with inhibitors of proteins that are highly or selectively expressed in SCLC, including PARP, already discussed in previous paragraph, and fucosyl-GM1.

### Fucosyl-GM1

Fucosyl-GM1 is a monosialoganglioside with limited expression in normal tissue, but highly expressed on SCLC cells. Encouraging preclinical results of BMS-986012 [161], a monoclonal antibody that binds to Fucosyl-GM1 with high affinity and specificity, led to a phase 1/2 clinical trial. In this trial the safety and preliminary efficacy of BMS-986012, both as a monotherapy and in combination with nivolumab, in patients with relapsed or refractory SCLC were evaluated [162]. Considering that the study enrolled pre-treated patients, albeit some without ICIs, the BMS-986012 plus nivolumab combination showed encouraging results in this first-in-human study with an ORR of 38% and a median OS of 18.7 months. Based on these results, a phase 2 study is currently evaluating the safety and efficacy of BMS-986012 combined with carboplatin, etoposide, and nivolumab in first line (NCT04702880).

Finally, one of the major areas of study and interest in the field of immunotherapy is chimeric antigen receptor (CAR) T cells, which are genetically modified T lymphocytes with a transgenic receptor capable of binding a specific antigen as well as an intracellular signalling domain that triggers the cytolytic effects. Initially, CAR T cells therapy was developed for blood malignancies but in recent years its use is being trialled for solid tumours, with encouraging results, even if they are less significant than haematological malignancies, which is due to tumour heterogeneity and hostile TME[163]. Different CAR-T cells have been developed against SCLC with promising results in pre-clinical studies. DLL3-CAR T cells showed preclinical positive results in term of safety and efficacy [164]. Anti-CD56 CAR T cells significantly reduced tumour burden in animal models of neuroblastoma and SCLC, but they only had a modest effect on survival; further investigations to limit toxicity related to on-target, off-tissue effects are needed [165]. AC133-specific CAR T cells exhibited an important cytotoxicity, and prolonged survival in a humanized orthotopic SCLC model, with a modest activity in monotherapy and a higher efficacy when combined with PD-1-inhibition and CD73-inhibition [166]. GD2-specific CAR T cells reduced the tumour burden in vitro and in vivo in xenograft models of GD2-expressing lung tumours (SCLC and NSCLC), and the susceptibility of tumours to this treatment was enhanced by pre-treatment with tazemetostat (EZH2 inhibitor), which can upregulate GD2 expression in tumour cells [167].

A phase 1 study to assess the tolerability and safety of the AMG 119, an anti-DLL-3 CAR T, was initiated in 2018, but it has been suspended for the time being (NCT03392064). A phase 1 first-in-human study with DLL3-targeted chimeric antigen receptor T cells (LB2102) and a phase 1 with autologous CAR T cells against the GD2 antigen in patients with advanced lung cancer, NSCLC and SCLC, are being planned (NCT05680922 and NCT05620342). So far, CAR-T cells have shown promising results in preclinical studies, but many more studies in-humans are needed before we can consider them an innovative therapeutic strategy in SCLC.

## Conclusions

Recently, many studies have led to the identification of the specific genetic abnormalities that characterise SCLC. The growing dataset in gene expression profiling in SCLC seems to be suggesting a new direction. A deeper understanding of SCLC at molecular level is at the base for the development of new, effective, and safe treatments. Several encouraging therapeutic approaches have been reported, the most promising of which target angiogenesis and epigenetics. Finally, therapies targeting proteins or oncogenic drivers show positive preliminary results (Table 2). Further studies based on molecular classification of SCLC and updated data on these novel therapies will hopefully improve outcomes in SCLC. Nonetheless, more research is needed to face the currently dismal prognosis and prospects of SCLC.

**Table 2:** Synoptic review of the most relevant clinical trials with new emerging agents in advanced SCLC

Target	Class and drug	Paper	Phase	Primary end point	Main result
DNA damage repair pathway					
	PARP-inhibitors				
	Olaparib + Temozolomide	Farango AF (30)	II	ORR	ORR 41.7%
	Olaparib	Woll P (32)	II	PFS	PFS 7.8 months
	Olaparib + Durvalumab	Thomas A (38)	II	ORR	ORR 10.5%
	Veliparib/Placebo + Temozolomide	Pietanza MC (31)	II	Improvement in 4-month PFS	PFS TMZ/veliparib (36%) vs TMZ/placebo (27%; p 0.19)
	Veliparib + Carboplatin + Ethoposide	Byers. LA (34)	II	PFS	PFS 5.8 months
	Niraparib	Aix SP (35)	III	PFS and OS	PFS 1.54 months and OS 9.92 months
	Lurbinectedin + Doxorubicin	Aix SP (44)	III	OS	OS 8.6 months
Angiogenesis					
	Bevacizumab + Cisplatin + Ethoposide	Tiseo M (56)	III	OS	mOS 6.7 months
	Pazopanib	Sun JM (58)	II	PFS	mPFS 3.7 months
	Pazopanib	Koinis F (59)	II	PFS-R at week 8	PFS-R 59% in platinum-sensitive cohort
	Sunitinib	Spigel DR (60)	II	1-year overall survival (OS)	1-year overall survival (OS) 54%
	Sunitinib	Ready NE (61)	II	PFS	mPFS 3.7 months
	Nintedanib	Han JY (62)	II	ORR	ORR 5%
	Anlotinib + platinum-based CT + Ethoposide	Liu C (65)	II	PFS	PFS 6.0 months
Protein and oncogenic drivers					
DLL-3	Rova-T	Blackhall F (82)	III	OS	mOS 6.3 months
	Rova-T	Johnson ML (83)	III	OS and PFS	OS 8.5 months, PFS not performed
	Tarlatamab	Paz-Ares L	I	Safety	Serious adverse events (AE) 51.4%
CD56	Lorvotuzumab mertansine	Socinski MA (88)	I/II	Phase I: dose-limiting toxicities (DLTs); Phase II: PFS	DLTs 21%, PFS 6.2 months
TROP2	Sacituzumab Govitecan	Bardia A (91)	I/II	Phase I: safety, phase II: ORR	Grade ≥ 3 TRAEs 59.6%, ORR 17.7%
SOX2	Sonidegib + Cisplatin + Ethoposide	Pietanza MC (93)	I	Safety	Serious AE 67%
	Vismodegib + Cisplatin + Ethoposide	Belani CP (94)	II	PFS and OS	PFS 4.4 months, OS 9.8 months
Bcl-2	Navitoclax	Rudin CM (96)	II	Safety	Dose interruption 46.3%
	Obatoclax mesylate + Carboplatin + Ethoposyde	Langer CJ (99)	II	ORR	ORR 62%
AURK	Alisertib + Paclitaxel	Owonikoko TK (105)	II	PFS	PFS 3.32 months
	Danusertib	Schöffski P (107)	II	Progression-free rate (PFR) at 4 months	PFR 0%
	Prexasertinib	Byers LA (108)	II	ORR	ORR platinum-sensitive 5.2%, platinum-refractory 0%
MYC	ADI-Peg 20			Ongoing trials	
Epigenetics					
HDAC inhibitors	Belinostat + Cisplatin + Ethoposide	Balasubramaniam S (133)	I	Maximum tolerated dose	Well tolerated. SCLC ORR: 43% SD, 57% PR
	Panobinostat	De Marinis F (134)	II	ORR	ORR 0%
	Romidepsin	Otterson GA (135)	II	ORR	ORR 0%
LSD1	GSK2879552	Bauer TM (140)	I	safety	Withdrawal 24%, 1 AE-related death
Overcoming the ICI resistance					
TIGIT	Tiragolumab + CT/ICIs	Rudin CM (160)	III	OS and PFS	OS 13.6 months (HR 1,04); PFS 5.4 months (HR 1,11)
LAG3	LAG525 (± spartalizumab)	Schöffski P (164)	I/II	Safety	Serious AEs: 5% mono, 5.8% combination. ORR 10% with combination
TIM3	Sabatolimab (± spartalizumab)	Curigliano G (168)	I/Ib	Safety	Grade ≥ 3 TRAEs 51%. ORR 6% with combination. AE G3-G5
Fucosyl-GM1	BMS-986012 (± Nivolumab)	Chu Q (170)	I/II	Safety	Grade 4 TRAEs 2%. ORR 4% mono, 38% with combination
Car-T				Ongoing Trials	
